# Racial Differences in Dermatologic Treatment for Adolescents With PCOS‐Related Acne Vulgaris

**DOI:** 10.1111/pde.70153

**Published:** 2026-02-16

**Authors:** Samantha Garcia, Brandi Kenner‐Bell

**Affiliations:** ^1^ Northwestern University Feinberg School of Medicine Chicago Illinois USA; ^2^ Division of Pediatric Dermatology Ann & Robert H. Lurie Children's Hospital of Chicago Chicago Illinois USA

**Keywords:** acne vulgaris, combined oral contraceptives, diversity, equity, and inclusion, health equity, hormonal therapy, polycystic ovary syndrome, spironolactone

## Abstract

**Background/Objectives:**

Polycystic ovary syndrome (PCOS) is a common endocrine disorder in women, with acne frequently appearing during adolescence. Racial and ethnic disparities in PCOS phenotype and adult acne treatment are well documented, yet differences in acne management among adolescents with PCOS remain unclear. This study evaluated racial and ethnic differences in acne treatment patterns among adolescents with PCOS.

**Methods:**

A retrospective chart review was conducted of female patients younger than 18 years with PCOS and acne vulgaris evaluated at a single urban academic medical center from 2012 to 2022. Demographic, clinical, and treatment data were extracted from the electronic health record. Group differences were assessed using *χ* ^2^ or Fisher's exact tests. Multivariable logistic regression models examined associations between race/ethnicity and receipt of acne therapies, adjusting for acne severity, insurance status, and interpreter use.

**Results:**

Among 138 adolescents (35.5% White, 10.9% Black, 7.9% Asian, 36.2% Hispanic, 9.4% Other), most had moderate acne (52.2%). Insurance type and interpreter use differed by race/ethnicity (*p* < 0.001). Hispanic patients had lower odds of spironolactone (aOR 0.22), combined oral contraceptives (COC) (aOR 0.16), and isotretinoin (aOR 0.08) prescription. Black patients had lower odds of topical antibiotic prescription (aOR 0.10).

**Conclusion:**

Racial and ethnic disparities exist in acne treatment among adolescents with PCOS, particularly reduced prescribing of spironolactone, COC, and isotretinoin for Hispanic patients and topical antibiotics for Black patients. These differences are not fully explained by acne severity, insurance, or interpreter use. Further research should assess how prescribing differences impact outcomes and inform equitable dermatologic care.

## 1 Introduction

Polycystic ovary syndrome (PCOS) is characterized by a combination of clinical signs and/or biochemical hyperandrogenism and ovulatory dysfunction persisting at least 2 years after menarche [1]. It is one of the most common endocrine disorders in women, affecting up to 15% of reproductive‐aged women and is the leading cause of infertility [2]. Its disease‐related disability score is comparable to or exceeding that of heart disease, diabetes, and breast cancer [2, 3]. Cutaneous manifestations of PCOS include acne vulgaris, hirsutism, acanthosis nigricans, and hidradenitis suppurativa. While the median age of diagnosis is in the late 20s, dermatologic manifestations such as acne frequently appear earlier, and both prevalence and severity of acne are higher when PCOS is diagnosed during adolescence compared to adulthood [4, 5, 6].

Racial and ethnic differences exist in PCOS prevalence and severity among pediatric patients. PCOS is more prevalent among Asian/Pacific Islander and Hispanic adolescents compared to non‐Hispanic White and Black adolescents [7]. South Asian and Hispanic adolescents tend to have more severe hyperandrogenic and metabolic features [8]. However, data specific to acne severity across ethnic groups among adolescents is limited [9].

Disparities also extend to general acne management. White patients are more likely to be prescribed oral antibiotics and combined oral contraceptives (COCs) than Black, Hispanic, and Asian patients [10, 11]. In addition, White patients are more frequently prescribed isotretinoin compared to Black and other non‐White counterparts [10, 11, 12]. There is mixed data on differences in prescription rates of spironolactone, topical antibiotics, and topical retinoids across race and ethnicity.

To our knowledge, no study has specifically evaluated differences in acne management by race and ethnicity among adolescents with PCOS. This represents a critical gap in the literature, as adolescents with PCOS experience a higher prevalence and severity of acne than their healthy peers, while also facing unique hormonal and metabolic factors that may influence treatment decisions [5]. Understanding whether treatment disparities extend to this population is essential for equitable dermatologic care.

## 2 Materials and Methods

### 2.1 Study Design and Population

This study was a retrospective chart review of female patients younger than 18 years who were evaluated between January 1, 2012, and December 31, 2022 at a single urban academic medical center. Eligible patients had documented diagnoses of both PCOS and acne vulgaris, confirmed using International Classification of Diseases (ICD) codes.

Patients were excluded if they did not have a confirmed diagnosis of PCOS and acne vulgaris, or if there was insufficient clinical documentation or data pertaining to acne parameters or treatment.

This study was approved by the institutional review board with waiver of informed consent given its retrospective design.

### 2.2 Data Collection

Data were abstracted from the electronic health record (Epic Systems) and managed in REDCap. Demographic variables included self‐reported race/ethnicity, age at diagnosis, insurance type (private vs. public), interpreter use, acne severity, and whether patients saw a pediatric dermatologist. Acne severity was determined from provider documentation when available. If not explicitly stated, severity was assigned using lesion‐based criteria based on documented physical exam findings: mild (presence of comedones only), moderate (presence of papules/pustules), and severe (presence of nodules, cysts, or scarring).

Treatment data included prescription of topical retinoids, topical antibiotics, oral antibiotics, spironolactone, COCs, and isotretinoin. Because COCs were often prescribed for both acne and PCOS, and these diagnoses frequently co‐occurred in provider documentation, it was not possible to reliably determine the primary indication for COC prescription between only acne or only PCOS. To address this limitation, prescriber specialty was recorded and used as a proxy for clinical indication.

### 2.3 Statistical Analysis

Demographic and clinical characteristics were described using counts and percentages. Categorical variables were compared across racial/ethnic groups using *χ* ^2^ or Fisher's exact tests, as appropriate.

To examine associations between race/ethnicity and specific acne treatments, odds ratios (ORs) were estimated using logistic regression. Multivariable models adjusted for key confounders selected a priori based on clinical relevance and prior literature, including acne severity, insurance type, and interpreter use. Results are reported as adjusted ORs (aORs) with 95% confidence intervals (CIs). To visually display disparities, forest plots of adjusted odds ratios for spironolactone and topical antibiotic prescription by race/ethnicity were generated.

Missing data for acne severity (8%) were handled by creating an “unknown” category to retain patients in analyses. Sensitivity analyses excluding patients with unknown severity produced similar results (data not shown).

All statistical tests were 2‐sided with *α* = 0.05. Analyses were conducted using R version 4.2.2 (R Foundation for Statistical Computing, Vienna, Austria).

## 3 Results

### 3.1 Demographic Characteristics

A total of 138 adolescents met inclusion criteria: White 35.5%, 49/138; Black 10.9%, 15/138; Asian 8.0%, 11/138; Hispanic 36.2%, 50/138; and Other 9.4%, 13/138 (Table 1). Most patients presented with moderate acne (52.2%, 72/138); severity did not differ by race/ethnicity (*p* = 0.49). Insurance type differed significantly by race/ethnicity (*p* < 0.001). Private insurance was most common among White patients (75.5%, 37/49), compared with Black (40.0%, 6/15), Asian (54.5%, 6/11), and Hispanic patients (26.0%, 13/50). Interpreter use was more common among Hispanic patients (38.0%, 19/50), compared with Asian patients (9.1%, 1/11), and was not observed among White (0%, 0/49), Black (0%, 0/15), or Other patients (0%, 0/13) (*p* < 0.001).

**TABLE 1:** Demographic characteristics of the study patients.

Characteristic	Race/ethnicity						*p*
	Non‐Hispanic White (*n* = 49)	Non‐Hispanic Black (*n* = 15)	Non‐Hispanic Asian (*n* = 11)	Hispanic (*n* = 50)	Other (*n* = 13)	Overall (*N* = 138)	
Acne severity							0.49
Mild	11 (22.4%)	6 (40%)	3 (27.3%)	7 (14%)	4 (30.8%)	31 (22.5%)	
Moderate	27 (55.1%)	4 (26.7%)	6 (54.5%)	29 (58.0%)	6 (46.2%)	72 (52.2%)	
Severe	7 (14.3%)	2 (13.3%)	2 (18.2%)	11 (22%)	2 (15.4%)	24 (17.4%)	
Unknown	4 (8.2%)	3 (20.0%)	0 (0%)	3 (6%)	1 (7.7%)	11 (8%)	
Insurance type							< 0.001*
Private	37 (75.5%)	6 (40%)	6 (54.5%)	13 (26%)	9 (69.2%)	71 (51.4%)	
Public	12 (24.5%)	9 (60%)	5 (45.5%)	37 (74%)	4 (30.8%)	67 (48.6%)	
Use of interpreter							< 0.001*
Yes	0 (0%)	0 (0%)	1 (9.1%)	19 (38%)	0 (0%)	20 (14.5%)	
No	49 (100%)	15 (100%)	10 (90.1%)	31 (62%)	13 (100%)	118 (85.5%)	
Seen by a dermatologist at least once	22 (44.9%)	4 (26.7%)	6 (54.5%)	22 (44%)	5 (38.5%)	59 (42.8%)	0.7

* Significant at *p* = 0.05 (Wilcoxon rank sum or Fisher's exact/Pearson's *χ* ^2^).

Overall, 42.8% (59/138) of patients were evaluated by a dermatologist at least once, with no significant differences by race/ethnicity (*p* = 0.7).

### 3.2 Treatment Patterns

Across the full cohort, the most frequently prescribed therapies were topical retinoids (80.0%, 110/138), combined oral contraceptives (86.2%, 119/138), and topical antibiotics (63.0%, 87/138) (Table 2). In unadjusted analyses, spironolactone prescribing differed significantly by race/ethnicity (*p* = 0.019). Spironolactone was prescribed to 38.8% (19/49) of White patients, 26.7% (4/15) of Black patients, 45.5% (5/11) of Asian patients, and 12.0% (6/50) of Hispanic patients. Isotretinoin prescribing also differed significantly by race/ethnicity (*p* = 0.045). Isotretinoin was prescribed to 5.1% (7/138) of White patients, 0% (0/15) of Black patients, 9.1% (1/11) of Asian patients, and 2.0% (1/50) of Hispanic patients.

**TABLE 2:** Treatment pattern characteristics.

Characteristic	Topical retinoid		Topical antibiotic		Oral antibiotic		Spironolactone		Oral contraceptive		Isotretinoin	
	No. (%)	*p*	No. (%)	*p*	No. (%)	*p*	No. (%)	*p*	No. (%)	*p*	No. (%)	*p*
Race/ethnicity		0.2		0.059		0.4		0.019*		0.2		0.045*
Non‐Hispanic White	35 (36%)		25 (40%)		21 (41%)		19 (53%)		46 (39%)		7 (70%)	
Non‐Hispanic Black	7 (7.1%)		2 (3.2%)		3 (5.9%)		4 (11%)		11 (9.2%)		0 (0%)	
Hispanic	38 (39%)		25 (40%)		17 (33%)		6 (17%)		41 (34%)		1 (10%)	
Non‐Hispanic Asian	7 (7.1%)		6 (9.7%)		5 (9.8%)		5 (14%)		10 (8.4%)		1 (10%)	
Non‐Hispanic Other/Multiple	11 (11%)		4 (6.5%)		5 (9.8%)		2 (5.6%)		11 (9.2%)		2 (20%)	
Acne Severity		0.003*		< 0.001*		0.024*		0.013*		0.012*		0.006*
Mild	17 (17%)		5 (8.1%)		6 (12%)		5 (14%)		29 (24%)		0 (0%)	
Moderate	58 (59%)		41 (66%)		28 (55%)		20 (56%)		61 (51%)		4 (40%)	
Severe	19 (19%)		14 (23%)		14 (27%)		11 (31%)		23 (19%)		6 (60%)	
Unknown	4 (4.1%)		2 (3.2%)		3 (5.9%)		0 (0%)		6 (5.0%)		0 (0%)	
Insurance type		0.5		0.4		0.024*		0.001*		> 0.9		0.3
Private	53 (54%)		35 (56%)		33 (65%)		27 (75%)		62 (52%)		7 (70%)	
Public	45 (46%)		27 (44%)		18 (35%)		9 (25%)		57 (48%)		3 (30%)	
Use of Interpreter	12 (12%)	0.2	8 (13%)	0.6	4 (7.8%)	0.089	2 (5.6%)	0.076	16 (13%)	0.5	0 (0%)	0.4

* Significant at *p* = 0.05 (Wilcoxon rank sum or Fisher's exact/Pearson's *χ* ^2^).

Treatment varied by acne severity. Patients with severe acne were more likely than those with mild acne to receive oral antibiotics (27.0%, 14/52 vs. 12.0%, 6/50; *p* = 0.024), spironolactone (31.0%, 11/52 vs. 14.0%, 5/50; *p* = 0.013), and isotretinoin (60.0%, 6/10 vs. 0%, 0/31; *p* = 0.006). Patients with private insurance were more likely to receive spironolactone (75.0%, 27/36 vs. 25.0%, 9/36; *p* = 0.001) and oral antibiotics (65.0%, 33/51 vs. 35.0%, 18/51; *p* = 0.024).

Distribution of combined oral contraceptive prescribers did not differ significantly by race/ethnicity (all *p* > 0.3). Most COCs were prescribed by primary care (56%–73% across groups), followed by endocrinology (27%–43%), with dermatology accounting for ≤ 7% (*p* values > 0.3).

### 3.3 Multivariable Analyses

**FIGURE 1:**
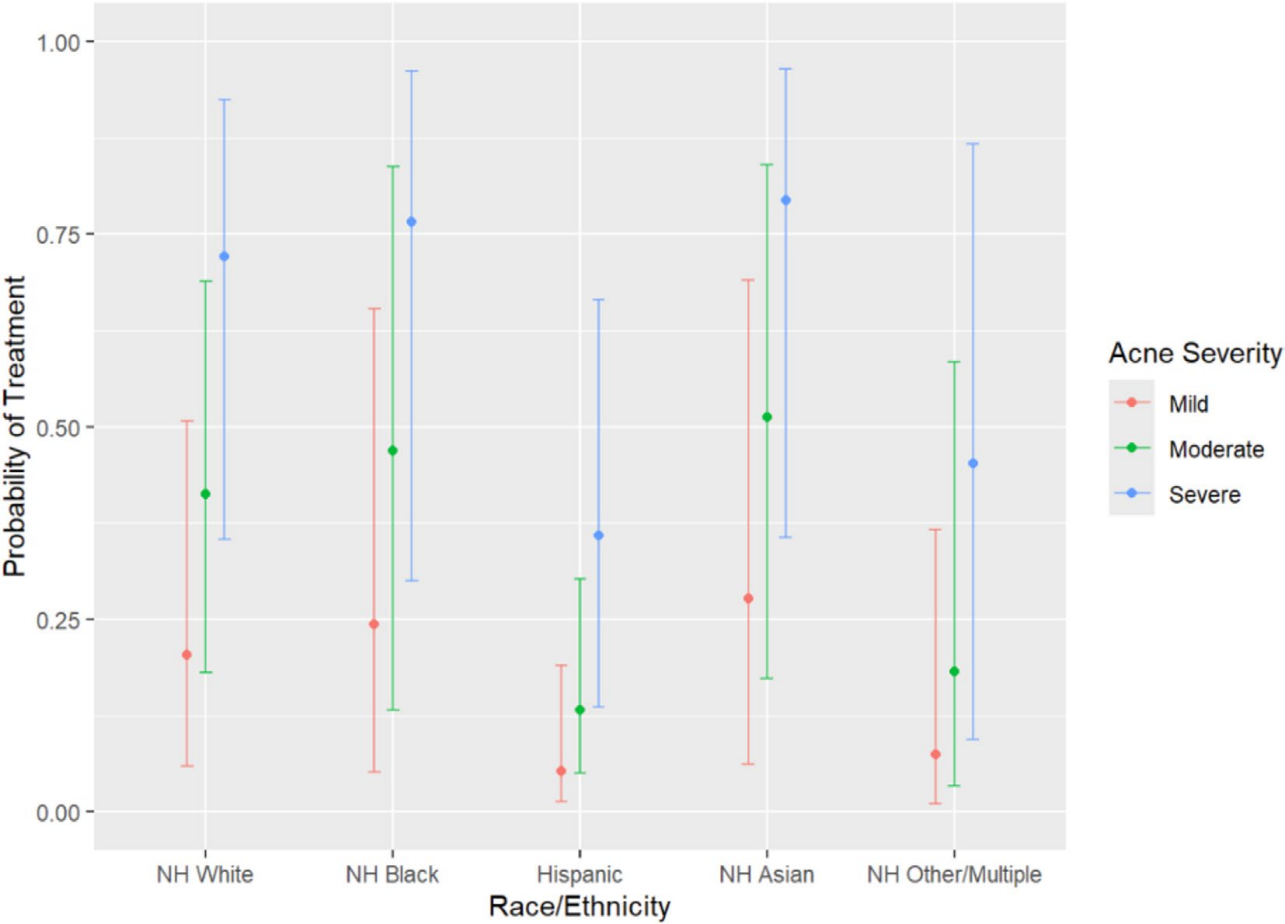
Adjusted odds ratio of spironolactone treatment by race and ethnicity.

**FIGURE 2:**
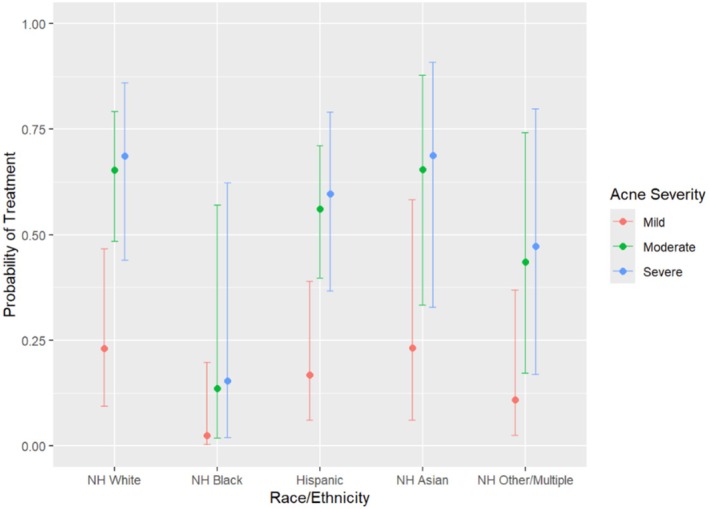
Adjusted odds ratio of topical antibiotic treatment by race and ethnicity.

After adjustment for acne severity, insurance type, and interpreter use, differences in treatment patterns persisted (Table 3). Hispanic patients had significantly lower odds of spironolactone (aOR 0.22, 95% CI 0.05–0.75; *p* = 0.021) (Figure 1) and COC prescription (aOR 0.16, 95% CI 0.02–0.86; *p* = 0.045) compared with White patients. Odds of isotretinoin use were also lower (aOR 0.08, 95% CI 0.00–0.68; *p* = 0.046). Black patients had significantly lower odds of receiving topical antibiotics compared to White patients (aOR 0.10, 95% CI 0.00–0.62; *p* = 0.037) (Figure 2).

**TABLE 3:** Adjusted ORs for specific acne prescription treatments.

Characteristic	Topical retinoid		Topical antibiotic		Oral antibiotic		Spironolactone		Combined oral contraceptive		Isotretinoin	
	Adjusted OR (95% CI)	*p*	Adjusted OR (95% CI)	*p*	Adjusted OR (95% CI)	*p*	Adjusted OR (95% CI)	*p*	Adjusted OR (95% CI)	*p*	Adjusted OR (95% CI)	*p*
Race/ethnicity												
Non‐Hispanic White	Reference		Reference		Reference		Reference		Reference		Reference	
Non‐Hispanic Black	0.34 (0.08, 1.40)	0.13	0.10 (0.00, 0.62)	0.037*	0.67 (0.12, 3.01)	0.6	1.26 (0.25, 6.09)	0.8	0.30 (0.02, 7.23)	0.4	0.00	> 0.9
Hispanic	2.54 (0.70, 11.0)	0.2	1.06 (0.37, 3.10)	> 0.9	1.12 (0.40, 3.17)	0.7	0.22 (0.05, 0.75)	0.021*	0.16 (0.02, 0.86)	0.045*	0.08 (0.00, 0.68)	0.046*
Non‐Hispanic‐ Asian	0.70 (0.16, 3.46)	0.6	1.17 (0.27, 5.53)	0.8	1.40 (0.32, 6.13)	0.6	1.50 (0.32, 7.10)		0.36 (0.03, 8.47)	0.4	1.19 (0.12, 8.62)	0.9
Non‐Hispanic Other/Multiple	1.88 (0.40, 13.8)	0.5	0.45 (0.10, 1.82)	0.3	0.79 (0.18, 3.07)	0.7	0.32 (0.04, 1.49)	0.2	0.19 (0.02, 1.85)	0.13	0.00	> 0.9
Acne severity												
Mild	Reference		Reference		Reference		Reference		Reference		Reference	
Moderate	2.94 (1.10, 7.97)	0.031*	6.43 (2.25, 21.7)	0.001*	2.64 (0.97, 8.11)	0.07	2.75 (0.89, 9.80)	0.094	0.41 (0.06, 1.80)	0.3	36,763,436 (0.00)	> 0.9
Severe	2.56 (0.73, 9.95)	0.2	7.59 (2.08, 32.0)	0.003*	6.50 (1.89, 25.0)	0.004*	10.1 (2.42, 50.1)	0.002*	1.65 (0.14, 38.8)	0.7	375,030,532 (0.00)	> 0.9
Insurance type												
Private	Reference				Reference		Reference		Reference		Reference	
Public	0.57 (0.21, 1.59)	0.3	0.52 (0.20, 1.29)	0.2	0.44 (0.17, 1.06)	0.072	0.24 (0.08, 0.67)	0.009*	2.50 (0.61, 13.0)	0.2	0.97 (0.15, 5.44)	> 0.9
Interpreter												
No	Reference		Reference		Reference		Reference		Reference		Reference	
Yes	0.39 (0.08, 1.73)	0.2	0.69 (0.18, 2.54)	0.6	0.63 (0.14, 2.51)	0.5	1.97 (0.23, 13.0)	0.5	0.70 (0.10, 4.67)	0.7	0.00	> 0.9

Abbreviation: OR, odds ratio.

* Significant at *p* = 0.05 (Fisher's exact/Pearson's *χ* ^2^).

Severe acne was a strong predictor of systemic therapy, including oral antibiotics (aOR 6.50, 95% CI 1.89–25.0; *p* = 0.004), spironolactone (aOR 10.1, 95% CI 2.42–50.1; *p* = 0.002), and COCs (aOR 10.1, 95% CI 2.42–50.1; *p* = 0.002).

## 4 Discussion

In this retrospective study of adolescents with acne in the setting of PCOS, significant racial and ethnic differences in dermatologic prescription patterns were identified. Hispanic patients were less likely than White patients to receive spironolactone, COCs, and isotretinoin. Insurance status and interpreter use were also important contextual factors. Hispanic patients were more likely to have public insurance and require interpreter services, aligning with broader disparities in healthcare access. However, adjustment for these variables did not fully account for treatment gaps, underscoring that language barriers and insurance limitations alone do not explain these differences in prescription patterns. Provider‐level factors, including differences in counseling approaches or implicit bias, may contribute. Patient‐level factors also play a role including differences in treatment‐seeking behavior and cultural perceptions of hormonal therapy. In some cases, spironolactone and COCs were discussed during visits but ultimately deferred by families and/or patients, reflecting preference‐sensitive decisions. These findings suggest that racial and ethnic differences in contraceptive attitudes and cultural beliefs—particularly during adolescence—may influence treatment decisions. All primary care, endocrinology, and dermatology provider groups routinely prescribe COCs as part of both PCOS and acne management, suggesting that observed differences are unlikely to be driven solely by provider availability or scope of practice.

Black patients were less likely to receive topical antibiotics. These differences persisted after accounting for acne severity, insurance status, and interpreter use, suggesting that factors beyond structural access barriers contribute to these differences. Lower topical antibiotic use among Black patients may reflect differential clinical prioritization, such as greater emphasis on treating post‐inflammatory hyperpigmentation with topical retinoids. Although topical retinoid use did not significantly differ by race/ethnicity in this cohort, we were unable to assess whether retinoids were preferentially selected for dual benefit in treating both active acne and hyperpigmentation; further qualitative studies are needed to clarify provider decision‐making.

Our results are consistent with prior work documenting disparities in adult acne treatment. Previous studies have shown that White patients are more likely to receive oral antibiotics, COCs, and isotretinoin compared to non‐White patients [10, 11, 12]. By focusing on adolescents with PCOS, our study extends this evidence to a population at greater risk for being affected by acne than non‐PCOS acne patients [5]. The underutilization of spironolactone and COCs among Hispanic patients is particularly concerning given their central role in treating hyperandrogenism, a key pathophysiologic mechanism in PCOS and acne.

While our study focused on prescribing patterns, an important next step would evaluate how these disparities translate into outcomes. Understanding whether racial and ethnic differences in access to spironolactone, COCs, or other therapies would help clarify whether inequities in prescribing directly contribute to long‐term dermatologic and psychosocial outcomes, including scarring and quality of life.

## 5 Strengths and Limitations

Strengths of this study include a racially and ethnically diverse cohort and adjustment for key confounders. However, several limitations warrant mention. First, the sample size of 138 patients, while sufficient to detect some statistically significant differences, limits power to identify smaller disparities and increases the possibility of type II error. With a larger population, additional differences may have emerged, and some marginal findings may have reached statistical significance. Second, acne severity classification is subject to variability: what providers document as mild, moderate, or severe may differ, particularly as not all treating clinicians were dermatologists. This introduces potential misclassification bias. Third, prescriber specialty was used as a proxy for the clinical indication for COCs. Because COCs were frequently prescribed for both acne and PCOS, and these diagnoses often co‐occurred in assessment and plan documentation, it was not possible to reliably distinguish acne‐directed prescribing from PCOS‐directed prescribing. To account for this, prescriber specialty was recorded as a proxy for indication, assuming that prescriptions from dermatology were more likely acne‐related, while those from endocrinology or primary care were more likely PCOS‐related. Even with this approach, overlap remained. This represents an important confounder that tempers the interpretation of disparities in COC prescription rates. In addition, reliance on provider documentation may undercapture discussions of therapy, clinical reasoning, or patient preferences.

## 6 Conclusion

Adolescents with PCOS‐related acne face significant racial and ethnic disparities in dermatologic treatment, with Hispanic patients less likely to receive spironolactone and Black patients less likely to receive topical antibiotics. While lower COC prescribing among Hispanic patients was observed, this finding is confounded by the dual indication of COCs for acne and PCOS, limiting its interpretation. These gaps persist beyond differences in severity, insurance, or prescriber specialty, underscoring deeper structural and provider‐level factors. Adolescence is a critical period to prevent scarring and psychosocial distress; addressing these inequities is essential. Future work should examine whether differences in prescribing translate into disparities in acne improvement, linking access to outcomes and guiding interventions to ensure equitable care for all adolescents.

## Conflicts of Interest

Dr. Kenner‐Bell: Advisory Boards for Dermavant and Pampers Serves on the speakers bureau for Regeneron Pharmaceuticals.

## Data Availability

The data that support the findings of this study are available from the corresponding author upon reasonable request.
